# A global dataset of air temperature derived from satellite remote sensing and weather stations

**DOI:** 10.1038/sdata.2018.246

**Published:** 2018-11-06

**Authors:** Josh Hooker, Gregory Duveiller, Alessandro Cescatti

**Affiliations:** 1 European Commission, Joint Research Centre, Directorate D–Sustainable Resources, Bio-Economy Unit, Ispra (VA) I-21027, Italy

**Keywords:** Climate change, Atmospheric dynamics, Applied mathematics, Climate and Earth system modelling

## Abstract

Air temperature at 2 m above the land surface is a key variable used to assess climate change. However, observations of air temperature are typically only available from a limited number of weather stations distributed mainly in developed countries, which in turn may often report time series with missing values. As a consequence, the record of air temperature observations is patchy in both space and time. Satellites, on the other hand, measure land surface temperature continuously in both space and time. In order to combine the relative strengths of surface and satellite temperature records, we develop a dataset in which monthly air temperature is predicted from monthly land surface temperature for the years 2003 to 2016, using a statistical model that incorporates information on geographic and climatic similarity. We expect this dataset to be useful for various applications involving climate monitoring and land-climate interactions.

## Background & Summary

Air temperature is a fundamental biophysical variable that influences almost all biotic processes, as well as many abiotic processes globally. Gridded climatologies describe how air temperature varies geographically and seasonally, but in reality there are very few datasets describing actual records of air temperature. What records there are tend either to describe only a limited number of locations (*e.g.* weather stations), or to be averaged over very large spatial resolutions as gridded products (*e.g.* CRUTEM4^1^ which is gridded at 5° spatial resolution).

Meteorological re-analysis products (*e.g.* ERA-Interim^2^ or ERA5) will provide gridded records of air temperature at high temporal resolution and at somewhat finer spatial resolutions than other gridded data products of air temperature, but these spatial resolutions can still represent fairly coarse gridcells that will mask local variation. What’s more, whilst re-analysis models are typically constrained by assimilated observations of temperature (and other physical variables), re-analysis values of temperature are still modelled (albeit from physically-informed meteorological models), and are not themselves observations.

Satellites monitor the Earth at relatively fine spatial and temporal resolutions, and measure radiant temperatures emitted from the Earth back into space. These observations are used to retrieve land surface temperature (LST^3^,), and gridded records are available that provide continuous estimates of LST in both space and time. Ideally, satellite retrievals of LST could be used to fill gaps of surface measurements of air temperature in both space and time. However, air temperature and LST are not identical, and it is not necessarily straightforward to develop robust estimates of air temperature from LST.

Various approaches by which air temperature might be derived from land surface temperature have been explored (*e.g*^4–9^.), with differing levels of success. These attempts have typically focused on limited geographic realms (e.g. within a given country or continent). Here, we develop a statistical model to predict air temperature from remotely sensed land surface temperature globally, and use it to estimate a novel dataset of monthly air temperature at spatial resolution of 0.05° for the interval 2003-2016.

## Methods

### General approach

Air temperature is fundamentally a spatio-temporal phenomenon, varying in both space and time. Adding to this complexity, temperatures vary at daily and seasonal scales. If we were to develop a model to predict air temperature for a given month, that month can represent either summer or winter conditions, depending on the particular location at which the prediction is to be made; this model would be expected to perform better if it were applied only to a given season. To cope with this issue, months in the two hemispheres can be re-aligned so that a prediction for (northern hemisphere summer) June, for example, is trained on both (northern hemisphere summer) June as well as (southern hemisphere summer) December. However, some places experience more summer-winter differences than others. For instance, the tropics experience a more continuous climate with respect to summer *vs.* winter; but they also experience more pronounced rainy *vs.* dry seasons, which will also affect the temperature regime. In order to develop a statistical model to predict air temperature at a given location and time of year, we therefore need to train the model with data appropriate for that location at that time of year (given all of the above). That would suggest using training data from only that location at that time of year, which is improbable because there is only a limited amount of training data.

To overcome this limitation we follow an alternative approach. Our objective is to predict time series of monthly air temperature for each gridcell in a global grid of remotely sensed land surface temperature observations. We therefore develop independent models for each month of the year, which bypasses the problem of training and predicting different months and seasons in the same model (there is little reason to expect the relationship between air temperature and land surface temperature to remain consistent over different seasons). This, however, still leaves the ‘geographical problem’, whereby training data from the same month can actually include different seasons (as the data come from different locations, or even, given that we are developing a global model, different hemispheres). To address this, we use geographically weighted regression (GWR^10,11^), so that predictions of air temperature at a given location and month are based on a ‘local’ model that is trained on ‘local’ observations. The training data are now ‘close’ in space (as we use GWR) and in time (as the model is developed for the individual month).

Predictions of air temperature are therefore made using statistical models that are trained on observations of air temperature from near to where those predictions are to be made. This assumes that ‘nearby’ implies some kind of consistency in the relationship between predictors and response. However, other measures or definitions of ‘near’ than geographic distance are possible, and so we also use an analogous regression where ‘near’ is instead defined by climatic similarity rather than geographic proximity. The rationale here is that within individual months, the relationship between predictor and response variables is more consistent amongst sites that experience similar climates, and that regression model coefficients can also vary in climate space.

**Figure 1:**
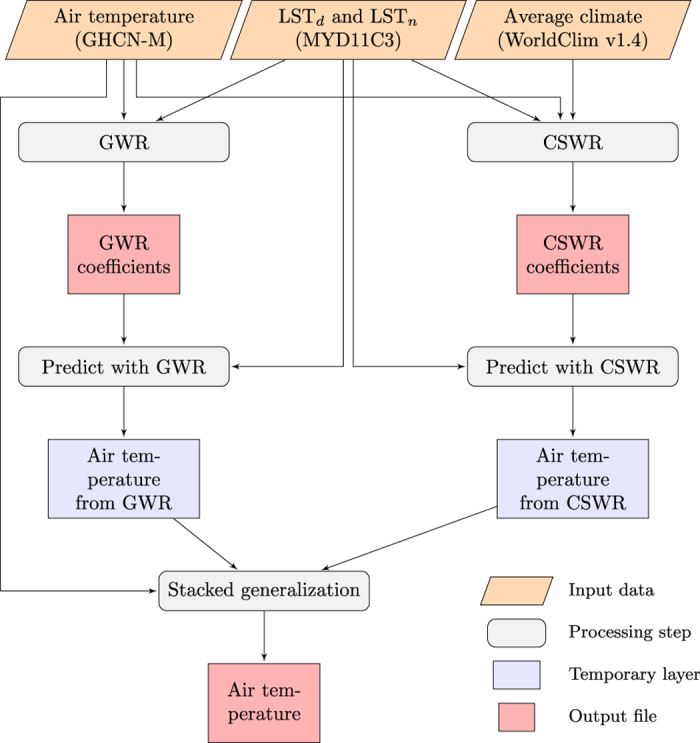
Schematic overview of the processing steps to generate the dataset. The different output files of the dataset are the air temperature and derived coefficients for both the geographically weighted regression (GWR), climate space weighted regression (CSWR) and the stacked generalisation (SG). LST_*d*_ and LST_*n*_ stand for daytime and nighttime land surface temperature respectively.

Following this approach we therefore develop geographically weighted regressions and climate space weighted regressions (CSWR) relating air temperature measured at weather stations to satellite-based observations of daytime and nighttime land surface temperature (*LST*_*d*_ and *LST*_*n*_ respectively). We then statistically combine predictions from these two models using a method called stacked generalisation^12^ to produce an ‘overall’ prediction of air temperature. Figure 1 provides a schematic overview of the processing steps used to generate this dataset.

### Input data

**Figure 2:**
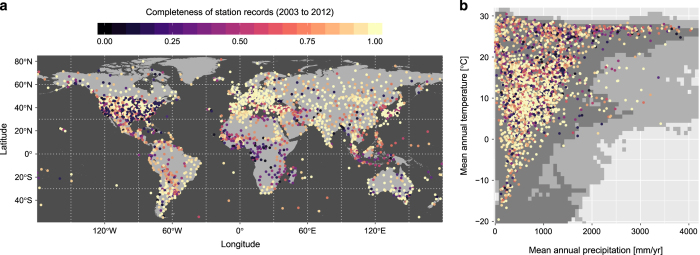
Location of the weather stations used to calibrate statistical relationships in this study. The level of completeness for each station during the calibration period 2003-2012 is reported according to (**a**) geographic location and (**b**) background climate. The climate space is defined based mean annual temperature and annually cumulated precipitation from WorldClim (version 1.4). The lightly shaded area behind the points show the complete range of values in WorldClim, while the darker area represents the those bins containing 99% of all records.

The Global Historical Climatology Network - Monthly (GHCN-M) dataset^13^ is used as the source for reference air temperatures. This dataset provides monthly average air temperature at a large number of weather stations from sometimes up to more than 100 years ago. The completeness of the record varies from station to station, and so a subset of 3253 stations containing at least one value during the period 2003-2012 is retained. Figure 2a maps the stations represented in this dataset, and also illustrates the completeness of each station’s coverage of the 2003-2012 period. While the distribution of these stations is not geographically uniform (there is a much denser coverage in the US and Europe), they largely cover most of the climate space, as defined by mean annual temperature and annually cumulated precipitation (Fig. 2b).

Remotely sensed measures of both daytime and nighttime LST are obtained from the MODIS instrument. Whilst this instrument is available on two platforms, Terra and Aqua, only the latter is used as its overpass (approximately 13:30 and 1:30 local time at the Equator) is closer to when diurnal minimum and maximum temperatures are expected. The MODIS land surface temperature algorithm provides estimates of daytime and nighttime LST at a monthly time step at 0.05° spatial resolution^3,14^ in the MYD11C3 product (we use collection 5 available from the NASA LPDAAC website https://lpdaac.usgs.gov/). While MODIS data is also available at finer temporal resolution (e.g. daily and 8-daily), and using these finer temporal data might ultimately lead to predictions of air temperature at the same revisit frequency, these data are much patchier due to cloud cover, and this can impede the estimation of LST. For this reason, we focus here on monthly values.

We use WorldClim version 1.4^15^ to define the monthly climate spaces over which CSWR is applied. This dataset provides the mean monthly temperature and mean monthly cumulated precipitation for the period 1960-1990 at a spatial resolution of 1/12°. A small minority of weather stations fall in WorldClim gridcells with no data (typically, Antarctica or small islands), and so these stations are excluded from all analyses (GWR as well as CSWR). There are therefore 3201 stations used in each analysis.

### Geographically weighted regression

When a regression is applied over geographically-distributed data, the coefficients of that regression model need not in fact to be constant over space. Geographically weighted regression was developed to deal with this non-stationarity. Rather than calibrating one regression for the whole domain, in GWR the model is instead fitted at each location within the domain of interest, but favouring training data from those locations that are spatially closer to the location at which the GWR is being applied. Practically, this is achieved by inversely weighting the observations in the training data given their distance from the location being analysed.

Given the global extent of our analysis, we use great circle distances to weight our GWRs. A negative exponential function is applied to these distances, so that training data from stations further away receive less weight:

(1)w=e−d2/l

(where *w* is the weight, *d* is the great circle distance between the location of analysis and training data observation, and *l* is a lengthscale that controls the rate of ‘distance decay’). This hyperparameter *l* must be specified before the GWR can be made, but there is usually no obvious or justifiable *a priori* value to use. We therefore use a jackknife (leave-one-out) procedure to assess a range of values: for each station we 1) develop a GWR for that station based on training data from all other stations’ (but not that station’s) observed air temperature records using the candidate values of *l*; 2) predict the air temperature values for that station; 3) repeat this for each station; 4) calculate the total root mean squared error between these predictions and the actual observations (RMSEP) over all stations for each candidate value of *l*; and, finally, 5) select the value of *l* that leads to the lowest RMSEP. We then use this value of *l* in the main GWR model.

We use the standard function to fit linear models in the R programming language, lm(), to fit these GWRs, providing each call of lm() with the appropriate vector of weights (the transformed distances to training data, as defined by equation 1) for the location being analysed. The specific form of the model is then:

(2)Tair=β0+β1×LSTn+β2×LSTd

The real result of the GWR is the derivation of these three *β* coefficients for all gridcells in the global domain (the coefficients differ across gridcells because each gridcell falls a different distance to each of the stations at which the training data have been measured). They can then be combined with respective observations of LST to predict air temperature in each gridcell globally. A separate GWR is applied for each month of the year, resulting in 12 models, and 12 sets of *β* coefficients.

### Climate space weighted regression

The logic of applying repeated weighted regressions based on proximity in geographic space can equally be extended to proximity in climate space. The relationship between air temperature and LST could even be more consistent over stations with similar climates, despite being separated geographically. CSWR is applied following exactly the same procedure as for GWR, except instead of using geographic coordinates to specify the distances and thus weights for the regressions, we instead now use the WorldClim climatology for each respective month to define a climate space. We use the WorldClim data with the spatial resolution that is closest to that of the satellite LST data. The variables used to define this climate space are average temperature and accumulated precipitation, which are measured on different scales and therefore need to be standardised (using the mean and standard deviation of each variable calculated over the locations of the weather stations) before Euclidean distances in this climate space are calculated between weather stations. These distances are then transformed using the same negative exponential function (equation 1), where the most appropriate lengthscale hyperparameter *l* is again identified through a jackknife leave-one-out process minimising RMSEP.

The ‘global’ model is then applied in the same way, except with the addition of one extra step: in estimating the *β* coefficients for each gridcell in the global domain, that gridcell’s monthly climatological average temperature and rainfall sum must first be looked up and standardised with the appropriate mean and standard deviation, before (Euclidean) climate space distances to training data locations (in climate space) are calculated and transformed. Again, a separate CSWR is applied for each month of the year, resulting in 12 models, and 12 sets of *β* coefficients.

### Coefficients and model predictions

**Figure 3:**
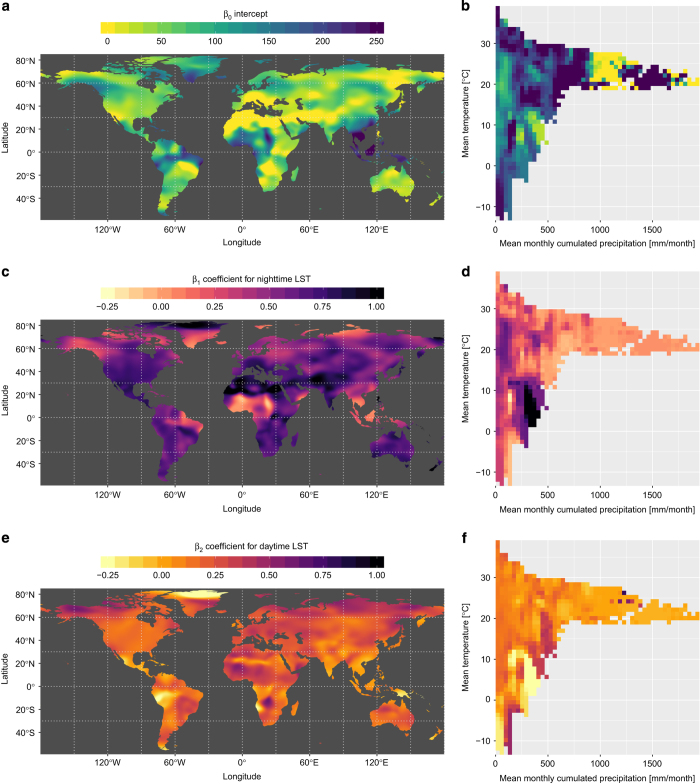
Regression coefficients for the month of June. The left column (**a**,**c** and **e**) maps coefficients for the geographically weighted regression (GWR), while the right column (**b**,**d** and **f**) presents the coefficients for the climate-space weighted regression (CSWR) in the (mean monthly temperature vs. mean monthly precipitation) climate space for the month of June.

Once both GWR and CSWR models are calibrated, a first result consists of the respective monthly *β* coefficients. Figure 3 shows these coefficients for the month of June, with the maps illustrating how the GWR *β* coefficients vary geographically, and the climate spaces illustrating how the CSWR *β* coefficients vary over climate space. These (monthly) coefficients can then be used to predict two sets of (monthly) maps of air temperature, one based on GWR and the other on CSWR.

### Stacked generalisation

Predictions of air temperature based on GWR and on CSWR are finally combined to make an overall prediction of air temperature, using stacked generalisation. Stacked generalisation is a method to optimally combine multiple statistical models into an ensemble model that will (theoretically, at least) lead to more accurate predictions than each of the individual ensemble members. More specifically, the individual ensemble members (which may each exhibit different forms of bias) are combined to minimise bias in the final model predictions and therefore improve model performance. In this case, the GWR is designed to remove the spatial structure in the residuals while the CSWR removes the climatic structure, and the stacked generalization then optimally combines both.

Essentially, an ensemble of models is *stacked*, where each of these models has been used to ‘jackknife predict’ each observation in the dataset (that is, each observation is predicted by the model trained on all the other observations less that observation), and then a *generalising* model of the actual observations is trained on the ensemble of stacked model predictions (which are now statistically independent of the observations themselves). We follow^16^ in using non-negative least squares to generalise, fitting the model:

(3)TSG=η1×TGWR+η2×TCSWR

where *T_SG_* is the stacked generalised prediction of air temperature, *T*_*GWR*_ and *T*_*CSWR*_ the GWR and CSWR jackknife predicted values of air temperature, and *η*_1_ and *η*_2_ are constrained to be non-negative coefficients, identified using the nnls() function from the nnls library for R.

Again, this model is applied to each month, and so 12 sets of coefficients are identified. These *η* coefficients reflect weighting factors between the GWR and CSWR models. They are constant across gridcells, and so only two exist for each month; they are reported in Table 1.

### Code availability

All computations have been done using the R environment for statistical computing version 3.4.3. An R code script named LST_2_AST.R is provided as supplementary material (Supplementary File 1). This code describes GWR and CSWR models, cross validates these models to find the best length scales for their respective weighting functions, and then combines GWR and CSWR models using stacked generalisation with non-negative least squares being used as the generalising model.

## Data Records

The entire dataset (Data Citation 1) consists of geo-localised gridded files and a table reporting information on the station data.

The first set of files consists of the final air temperatures resulting from the stacked generalization with a spatial resolution of 0.05 and a monthly temporal frequency. These data are stored in NetCDF format with one file per year, each defined by 3 dimensions (lat, lon and time, respectively representing latitude, longitude and the month of the year) and containing a single variable (Tair). Data are stored as signed 16 bit integers using a scale factor of 0.00275 and an offset of −282, both stored as attributes in the NetCDF file. File names follow this naming convention: Tair_SG_ <YYYY> .nc, where <YYYY > represents the year. This air temperature archive might also be considered in two parts. The first, covering the years 2003 to 2012, consists of predictions of air temperature based on models trained on observations of air temperature from the same period. The second, covering the years 2013 to 2016, consists of predictions of air temperature based on those same model coefficients, and so might be considered somewhat more ‘out-of-sample’.

We additionally provide the intermediate products of air temperature obtained from the GWR and CSWR methods. These follow the same file structure than those resulting from the stacked generalization (SG) described above, with only a change in the file name in which SG is replaced by either GWR or CSWR.

Yet another set of files is provided containing the calculated coefficients for both the GWR and the CSWR. There is one NetCDF file for each parameter of each regression type. Each file is defined by its 3 dimensions (lat, lon and month) and contains a single variable (either *β*_0_, *β*_1_ or *β*_2_), stored as signed 16 bit integers using individual scale factors and offsets, which are stored as attributes in their respective NetCDF files. File names follow this naming convention: Coeffs_ <RegType >_< Coef > .nc, where <RegType> denotes the regression type (GWR or CSWR) and <Coef> represents the coefficient (b0, b1 or b2).

The table containing the station data consists of a comma separate values (CSV) file with 3076 records of all stations used in this work. The columns provide information on the longitude (Lon) and latitude (Lat) of each station, the degree of completion of the data record from 0 to 1 (nObs), the mean temperature (meanT) and mean monthly precipitation (meanP) of the area where the station is according to WorldClim, the RMSEP of the final stacked generalized models (SG_rmsep), the RMSEP for the GWR model (G_rmsep), the RMSEP for the CSWR model (C_rmsep), and the relative importance of each RMSEP as calculated by equation 4 (PerMod_RMSEP).

## Technical Validation

The dataset we describe consists of predictions made from a statistical model that we have developed. Independent observations of air temperature, with which we might validate these predictions, are not available. However, the nature of our statistical model lends itself to cross-validation.

Both GWR and CSWR models are jackknifed to identify the most appropriate lengthscale hyperparameters for their respective weighting functions. This leads to RMSEP values for each model, representing the average error of prediction by each method when it independently predicts air temperature (by independently, we mean when air temperature is predicted using that model, but the observation whose value is being predicted is not being used in the training of that model). These RMSEP values are reported for both GWR and CSWR models for each month of the year in Table 2. This same table also reports the RMSEP values where a simple linear model has also been used to predict air temperature directly from LST. GWR and CSWR RMSEP values are always lower than linear model RMSEP values, indicating that these models show real improvement over the linear approach.

Such a cross-validation of stacked generalised predictions of air temperature is a little more complex. Whilst GWR and CSWR predictions are statistically independent of the observations against which they are assessed, stacked generalised predictions are not (at least, not completely). Even though the stacked predictors in the generalising model are independent of the observations, the observations are themselves then used to train the generalising model, and so any predictions arising from this model are not independent. If we were to leave an observation out of the generalising model, to then predict that observation with a generalising model trained without that observation, the prediction would still not be entirely independent of that observation, because that observation has been used to train of each of the other elements of the stacked predictors (site A is left out to predict site A, it is not left out to predict site B, when site B would then be used in the generalising step to predict site A again).

Instead, we undertake a stricter cross-validation where a site is left out, and a completely independent stacked generalised model is established to predict that site. That is, a GWR and a CSWR are derived without this site, where each of the other sites is in turn left out, to identify the best lengthscale *without that site*, and so that site has not been used in independently predicting any of these other sites. The resulting GWR and CSWR are then stacked generalised *still without that site*, and only then is that site predicted. This is repeated for all sites.

The RMSEPs resulting from this process are also reported in Table 2, where they can be seen to be consistently better again than the GWR and CSWR results. It is fairly safe to conclude that the stacked generalised model is better than both GWR and CSWR models, which are in turn better than the linear model.

Note that in all cross-validation assessments, whole sites (*i.e.* weather stations) have been left out, not just individual records of air temperature for a given month at a given location. This ensures that a stricter cross-validation is achieved, and one that better represents the ‘transference’ of the model between locations, which is the real aim of the model when we wish to predict air temperature across the globe.

**Figure 4:**
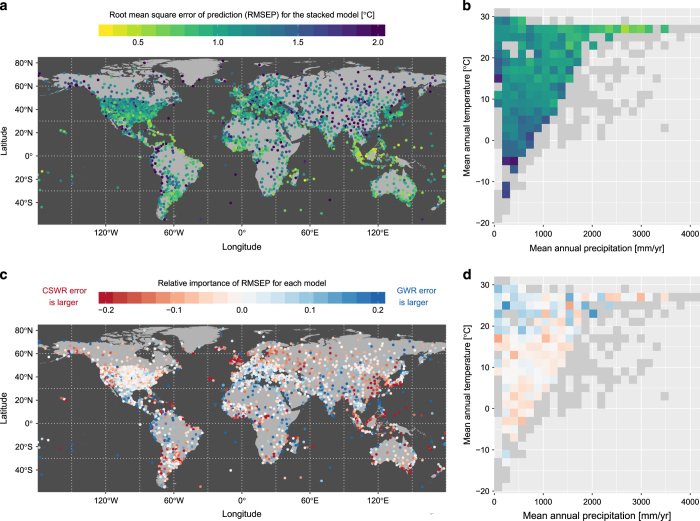
Prediction error in the estimation of air temperature. The top panel provides the root mean square error of prediction (RMSEP) at each station after the stacked generalization procedure declined in both (**a**) geographic and (**b**) climatic space. For the climatic space, the mean RMSEP is provided only for bins containing at least 5 stations, while the others remain greyed out. The bottom panel shows the relative contribution of the two individual models to the RMSEP again in (**c**) geographic and (**d**) climatic space, with red and blue respectively indicating whether the error is larger for the CSWR model or the GWR model.

The RMSEP values reported in Table 2 are averaged across all observations at all sites. We also plot the site-level RMSEP values from the stacked generalisation model in Fig. 4a (in geographic space) and Fig. 4b (in climate space). There are no major spatial patterns in the quality of the predictions. While stations in colder places seem to suffer slightly worse predictions, this may be due to the sparser representation of these climates in the observed data.

We also provide an analysis comparing the separate RSMEP for the two different models (GWR and CSWR). Figure 4c shows the relative importance of RSMEP for each model calculated as:

(4)RelRMSEP=RMSEPGWR−RMSEPCSWRRMSEPGWR+RMSEPCSWR

The patterns show a balanced outcome with no model being systematically more prone to error than the other. As expected, the GWR does show higher errors for warmer regions where there is generally a lower station density, but there are also many places in the tropics where the GWR provides higher accuracy. Areas with densely populated station networks are also not necessarily dominated by better performance when using the geographic model, with a particular contrast between Northern and Southern Europe. Overall these results show how both GWR and CSWR are complementary and this complementarity does not necessarily follow an obvious pattern.

**Figure 5:**
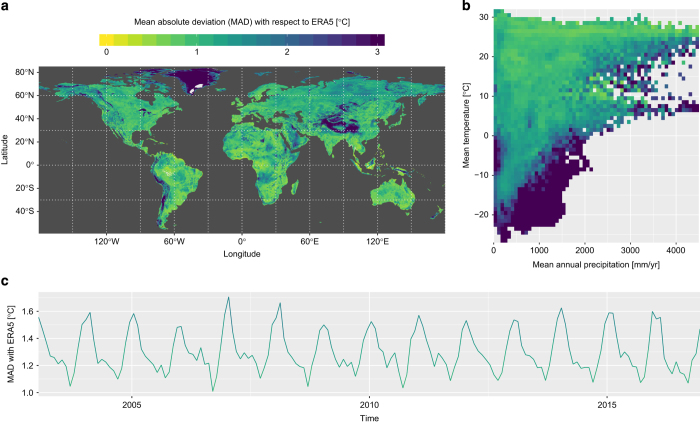
Inter-comparison with the air temperature from ERA5. The mean absolute deviation (MAD) between the air temperature values presented here and the air temperature provided by the ERA5 climate reanalysis is calculated per (**a**) spatial pixel, (**b**) climate bin, and (**c**) time frame; thereby illustrating respectively the agreement in time, in climate and in geographic space.

To complement this validation, an inter-comparison with 2 m air temperature from ERA5 reanalysis is provided in Fig. 5. To match both datasets together, the ERA5 hourly data is averaged to a monthly temporal resolution while our air temperature data is spatially aggregated from 0.05° to 0.25°. The comparison is first done by calculating the mean absolute deviation (MAD) between all *η* paired values of a given ensemble:

(5)MAD=∑i=1n|TSG,i−TERA5,i|

When considering the entire data cube all together, the MAD is 1.302°C. However, the MAD can also be calculated for different subsets, such as per time series, per climate bin, or per time frame, resulting in the respective declinations of MAD in Fig. 5a–c. The spatial representation of the temporal agreement (Fig. 5a) shows that the larger deviations occur in Greenland, the Tibetan plateau and the Andes. The deviations in climate space (Fig. 5b) are higher for colder and wetter areas, which correspond to places with less input information (relevant for both our method and for the ERA5 reanalysis). The time series of spatial deviations (Fig. 5c) shows there is a cyclic pattern, with smaller deviations in boreal summer, particularly in September, and a peak in January. It also shows there is no general trend in the deviations with ERA5. Analogous graphs reporting the overall agreement between the datasets using a dedicated index of agreement^17^ are reported in Supplementary Figure 1. As mentioned before, reanalysis data are not observations, and therefore this comparison with ERA5 does not serve as a proper validation; it is given here to provide broader context to the stacked generalised dataset.

## Usage Notes

We provide files with predicted air temperatures from 2003 to 2016. However, we also provide files with monthly coefficients for both GWR and CSWR models, and provide the stacked generalisation coefficients in Table 1 and in a separated file, meaning that air temperatures can be predicted independently if nighttime and daytime LST values are to hand.

The intermediate temperature values resulting from the individual GWR and CSWR models are provided in case they might be of use for any particular application. However, we recommend general users to prefer the final stacked generalized results (SG), as these should provide better estimates than either of the intermediate GWR and CSWR results. Users should not expect the data to be precise beyond the hundredth of a degree.

We used MODIS collection 5 LST data which is no longer updated operationally, and has instead been superseded by MODIS collection 6. We would expect little difference if MODIS collection 6 LST were used in place of collection 5 to predict air temperature, but cannot guarantee this.

From the analysis of Fig. 4a, and to a lesser extent Fig. 5a, it appears that the performance of our product is lower at higher elevation areas. As elevation is not explicitly taken into account in our modelling structure, and radiation load can differ for similar climates characterized only by mean temperature and precipitation, this may lead to higher uncertainty for such areas. Incorporating this effect could be envisaged in future improvements of this methodology, but in the meantime, users should consider values at higher elevation to be of lower quality.

## Additional information

**How to cite this article**: Hooker, J. *et al*. A global dataset of air temperature derived from satellite remote sensing and weather stations. *Sci. Data*. 5:180246 doi: 10.1038/sdata.2018.246 (2018).

**Publisher’s note**: Springer Nature remains neutral with regard to jurisdictional claims in published maps and institutional affiliations.

## Supplementary Material



Supplementary Figure S1

Supplementary File 1

## Figures and Tables

**Table 1:** Weighting coefficients for the stacked generalisation.

Month	GWR	CSWR
January	0.6002	0.3997
February	0.5691	0.4309
March	0.5655	0.4345
April	0.4320	0.5680
May	0.3490	0.6509
June	0.2861	0.7139
July	0.3407	0.6593
August	0.3780	0.6219
September	0.3080	0.6920
October	0.3332	0.6668
November	0.3790	0.6209
December	0.4904	0.5096
These ‘global’ numbers are used to combine predictions from geographically weighted regressions (GWR) and climate space weighted regressions (CSWR) for each month.		

**Table 2:** Root mean square errors of prediction (RMSEP) in C for linear regression (LM), geographically weighted regression (GWR), climate space weighted regression (CSWR) and the stacked generalised (SG) models of air temperature.

Month	LM	GWR	CSWR	SG
January	2.10	1.61	1.67	1.55
February	1.97	1.53	1.56	1.49
March	1.80	1.40	1.44	1.35
April	1.71	1.34	1.31	1.27
May	1.62	1.30	1.22	1.18
June	1.62	1.31	1.17	1.15
July	1.60	1.31	1.21	1.18
August	1.52	1.27	1.19	1.15
September	1.45	1.26	1.17	1.14
October	1.45	1.27	1.19	1.16
November	1.63	1.38	1.32	1.29
December	1.92	1.52	1.52	1.45
