# Supplementary material for: A global dataset of air temperature derived from satellite remote sensing and weather stations
Source: Sci Data. 2018 Nov 6;5:180246. doi: 10.1038/sdata.2018.246 (PMC6219417; doi:10.1038/sdata.2018.246)
Supplement: Supplementary Figure S1 [file sdata2018246-s2.pdf]

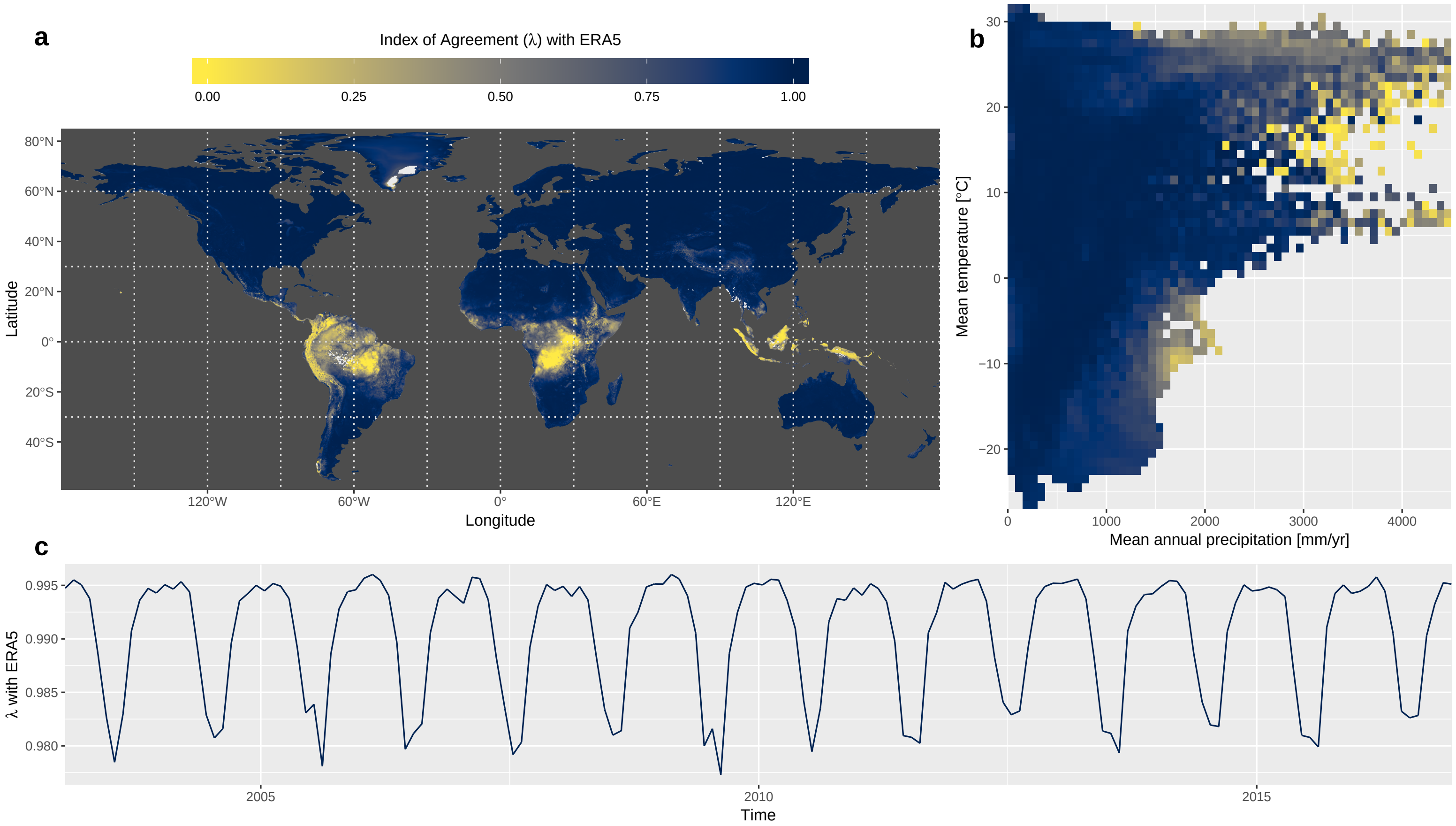

Supplementary Figure S1: **Agreement between the air temperature proposed in the present work with that of ERA5.** The index of agreement (λ) proposed by Duveiller et al. (2016) is used to characterise the range between no agreement ($\lambda = 0$) and total agreement ($\lambda = 1$) for the following subsets: (**a**) time series for each spatial pixel, (**b**) all across space and time values falling in a specific climate bin, and (**c**) a spatial values in a given time frame; thereby illustrating respectively the agreement in time, in climate and in geographic space.
